# Supplementary figures and images for: Exogenous Application of Glycine Betaine on Sweet Cherry Tree (Prunus avium L.): Effects on Tree Physiology and Leaf Properties
Source: Plants (Basel). 2022 Dec 11;11(24):3470. doi: 10.3390/plants11243470 (PMC9784700; doi:10.3390/plants11243470)

## Supplementary Materials

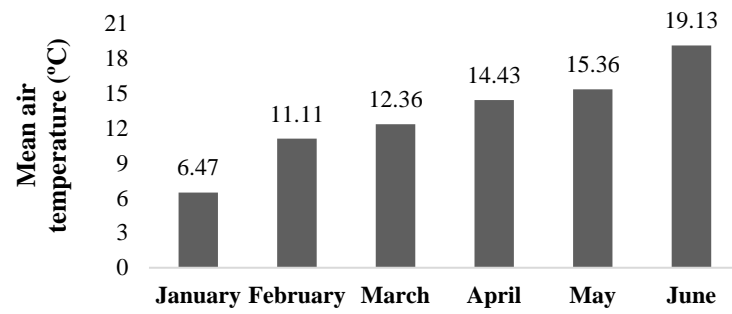

**Figure S1.** Mean air temperature (°C) from January to June of 2021.

Supplement: Supplementary file 1 [file plants-11-03470-s001.zip › plants-2053478-supplementary.pdf]
